# LDL Cholesterol Modulates Human CD34+ HSPCs through Effects on Proliferation and the IL-17 G-CSF Axis

**DOI:** 10.1371/journal.pone.0073861

**Published:** 2013-08-26

**Authors:** Thomas R. Cimato, Beth A. Palka, Jennifer K. Lang, Rebeccah F. Young

**Affiliations:** Department of Medicine/Division of Cardiovascular Medicine, State University of New York at Buffalo, School of Medicine and Biomedical Sciences, Clinical and Translational Research Center, Buffalo, New York, United States of America; Northwestern University, United States of America

## Abstract

**Background:**

Hypercholesterolemia plays a critical role in atherosclerosis. CD34+ CD45dim Lineage- hematopoietic stem/progenitor cells (HSPCs) give rise to the inflammatory cells linked to atherosclerosis. In mice, high cholesterol levels mobilize HSPCs into the bloodstream, and promote their differentiation to granulocytes and monocytes. The objective of our study was to determine how cholesterol levels affect HSPC quantity in humans.

**Methods:**

We performed a blinded, randomized hypothesis generating study in human subjects (n=12) treated sequentially with statins of differing potencies to vary lipid levels. CD34+ HSPC levels in blood were measured by flow cytometry. Hematopoietic colony forming assays confirmed the CD34+ population studied as HSPCs with multlineage differentiation potential. Mobilizing cytokine levels were measured by ELISA.

**Results:**

The quantity of HSPCs was 0.15 ± 0.1% of buffy coat leukocytes. We found a weak, positive correlation between CD34+ HSPCs and both total and LDL cholesterol levels (r^2^=0.096, p < 0.025). Additionally, we tested whether cholesterol modulates CD34+ HSPCs through direct effects or on the levels of mobilizing cytokines. LDL cholesterol increased cell surface expression of CXCR4, G-CSFR affecting HSPC migration, and CD47 mediating protection from phagocytosis by immune cells. LDL cholesterol also increased proliferation of CD34+ HSPCs (28 ± 5.7%, n=6, p < 0.03). Finally, the HSPC mobilizing cytokine G-CSF (r^2^=0.0683, p < 0.05), and its upstream regulator IL-17 (r^2^=0.0891, p < 0.05) both correlated positively with LDL cholesterol, while SDF-1 levels were not significantly affected.

**Conclusions:**

Our findings support a model where LDL cholesterol levels positively correlate with CD34+ HSPC levels in humans through effects on the levels of G-CSF via IL-17 promoting mobilization of HSPCs, and by direct effects of LDL cholesterol on HSPC proliferation. The findings are provocative of further study to determine if HSPCs, like cholesterol levels, are linked to CVD events.

## Introduction

Hypercholesterolemia is mechanistically tied to the pathogenesis of coronary artery disease, characterized by the formation of cholesterol and lipid rich plaques in the vessel wall invaded by immune and inflammatory cells [1]. Primary prevention of cardiovascular disease trials with HMG-CoA reductase inhibitors illustrate that reduction in cholesterol levels decreases cardiovascular disease events [2]. While it is recognized that hematopoietic cells are present in atherosclerotic plaques, their specific roles in coronary disease progression, and modulation by disease modifying therapies remain to be determined [3].

Undifferentiated hematopoietic stem/progenitor cells (HSPCs) migrate from the bloodstream into diseased tissue and differentiate to macrophages, monocytes, and neutrophils in response to infection, and inflammation [4]. HSPCs are also released into the bloodstream after acute myocardial infarction [5] and augment formation of new monocytes that participate in progression of atherosclerosis [6]. This indicates that circulating HSPCs in the bloodstream represents a pool of undifferentiated stem cells that play a role in the pathogenesis of coronary artery disease. However, the effects of hypercholesterolemia on HSPC numbers in the bloodstream of humans have not been determined.

Hypercholesterolemia causes elevation of leukocyte and monocyte counts in humans [7]. Several recent studies in normal mice fed high cholesterol diet [8], in LDLR ^-/-^ [9] or ABCA1^-/-^ ABCG1^-/-^ [10] cholesterol efflux transport knockout animals show that disordered cholesterol metabolism increases HSPCs in the bloodstream, and promotes their differentiation to myeloid and monocyte lineages that contribute to atheromatous plaque formation [11]. While these findings mechanistically tie cholesterol and cholesterol metabolism to hematopoietic stem cell mobilization and function, the cholesterol levels in hypercholesterolemic mice are roughly three times as high as those found in patients with acute coronary syndromes [12] and ten times the normal level of cholesterol in animals fed a normal diet [9]. Given this backdrop, the goal of this study was to determine the relationship between serum lipid levels and CD34+ HSPC levels across a range of cholesterol values commonly encountered in patients with acute coronary syndromes.

We performed a blinded, randomized observational study in human subjects with no history of cardiovascular disease events to determine the relationship between serum lipid levels and the circulating CD34+ HSPCs. We used statin therapy for two weeks duration (atorvastatin 80 mg daily, pravastatin 80 mg daily or rosuvastatin 10 mg daily) to vary the lipid levels from their baseline values and determined the quantity of CD34+ CD45dim Lineage- HSPCs in peripheral blood. The quantity of blood borne HSPCs significantly decreased with reduction of serum lipids, and regression analysis revealed the HSPC levels positively correlate with total and LDL cholesterol levels. The levels of HSPCs also correlated positively with G-CSF levels, and LDL cholesterol levels positively correlated with G-CSF levels and IL-17 levels in plasma. Our findings represent novel initial evidence to link cholesterol levels with the quantity of circulating HPCs through effects on the IL-17/G-CSF axis.

## Materials and Methods

### Patient Consent for Participation

Our research protocol was reviewed and approved by the University at Buffalo Intramural Review Board for Health Sciences research (Approval Number: MED5980509B). Informed consent to undergo the study protocol was obtained in writing from each participant, and conducted according to the principles expressed in the Declaration of Helsinki. Our study was organized as an observational trial as no direct health outcomes were to be measured. The study population comprised 12 adult subjects with no active medical problems. Study subjects were screened for the absence of chronic health disorders including hypercholesterolemia with cardiovascular risk factors, cancer, diabetes, chronic liver or kidney disease.

### Study Protocol

Study subjects were randomized to drug regimen groups using a block randomization design. The investigators were blinded to which treatment subjects were receiving. Study subjects underwent a baseline blood draw in which a complete blood cell count, lipid panel (total, HDL, and LDL cholesterol, and triglycerides) and C-reactive protein were determined by the Kaleida Health pathology laboratory. The baseline clinical characteristics of the study cohort are summarized in Table 1. Venous blood mononuclear cells were also obtained for flow cytometry analysis described below. Subjects were then treated for two weeks with one of three different HMG-CoA reductase inhibitors (pravastatin 80 mg daily, atorvastatin 80 mg daily, or rosuvastatin 10 mg daily). At the end of the two-week statin treatment, venous blood was sampled again to obtain the complete blood cell count, lipid panel, C-reactive protein level, and blood mononuclear cells for flow cytometry analysis. Subjects were then given a four-week statin free period. At the end of the four-week period venous blood was sampled again to determine the baseline blood counts, lipid profile, C-reactive protein levels, and blood MNCs. Then the next HMG-CoA reductase inhibitor in the randomization scheme was given for two weeks. The same protocol was repeated for statin drugs two and three until study completion.

**Table 1:** Clinical Data.

		**Mean Values ± SD**			**p-Value (statin vs. baseline)**		
	Baseline	Atorvastatin	Pravastatin	Rosuvastatin	Atorvastatin	Pravastatin	Rosuvastatin
**Age**	43.4 ± 12.5						
**BMI**	24.9 ± 7.2						
**Framingham Risk Score**	1.7 ± 0.5						
**Total Cholesterol (mg/dL)**	210.5 ± 27.6	138.5 ± 28.9	160.6 ± 28.9	154.2 ± 21.0	<0.0001	<0.0001	<0.0001
**LDL Cholesterol (mg/dL)**	136.2 ± 22.9	68.2 ± 12.1	88.7 ± 24	83.3 ± 12.7	<0.0001	<0.0002	<0.0001
**HDL Cholesterol (mg/dL)**	53.5 ± 12.9	54.1 ± 18.6	54.5 ± 13.1	55.3 ± 15.3	NS	NS	NS
**C-Reactive Protein (mg/L)**	1.1 ± 1.3	1.05 ± 1.1	0.95 ± 0.8	1.3 ± 1.5	NS	NS	NS
**CD34+ Lin- HSPCs**	0.15 ± 0.1	0.11 ± 0.06	0.1 ± 0.06	0.1 ± 0.04	<0.07	<0.02	<0.04
**CD34+ CD45dim HSPCs**	0.15 ± 0.1	0.1 ± 0.06	0.1 ± 0.06	0.09 ± 0.04	<0.06	<0.02	<0.05

### Definition of Risk Factors for Coronary Artery Disease

Risk factors for coronary artery disease were determined using the Framingham risk formula [13].

### Flow cytometry quantification of Hematopoietic Progenitor Cells (HPCs)

**Figure 1:**
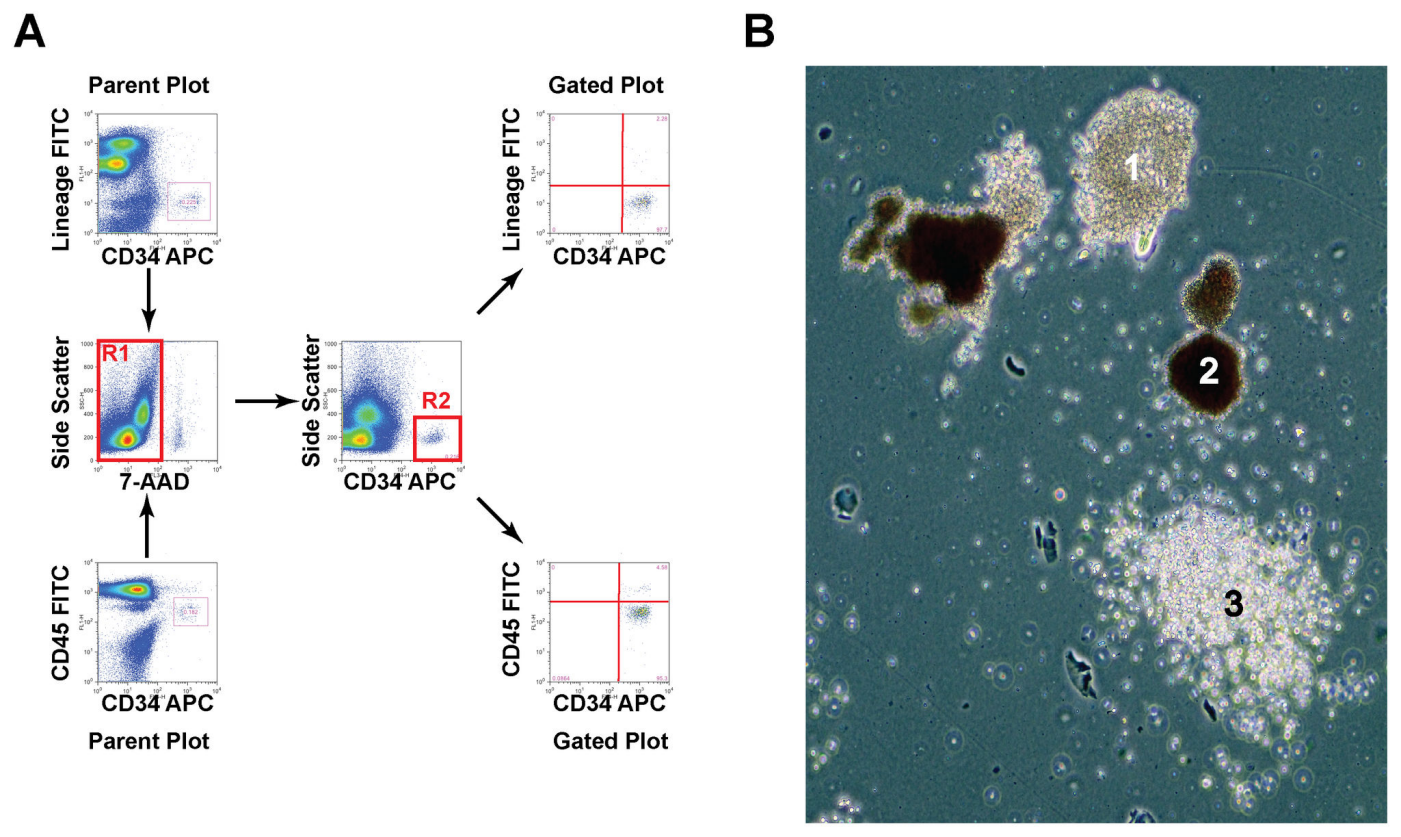
Gating Strategy and Differentiation of CD34+ Lineage- CD45dim Cells from Peripheral Blood Mononuclear Cells. Panel **A**: Representative flow cytometry plots and gating strategy to quantify CD34+ Lineage- CD45dim cells in human blood. CD34 CD45 or CD34 and Lineage cocktail plots (Parent Plots) were then gated to exclude dead cells (R1 gate), and positively select CD34+ cells low in side scatter (R2 gate). Panel **B**: Sorted CD34+ Lineage- CD45dim cells were differentiated in methylcellulose with cytokines to granulocyte (1), erythroblast (2), and monocyte colonies(3).

HPCs were quantified using the following approach: Venous blood samples were collected into BD Vacutainer CPT tubes, and then centrifuged at 1500 x g for 30 minutes. The mononuclear cell fraction was retained, and the MNCs were washed three times in phosphate buffered saline with 0.1% bovine serum albumin. MNCs were enumerated using a hemocytometer. 1 million cells were then stained with human lineage cocktail 1-FITC (BD Biosciences), or CD45-FITC (Clone J33, BD Biosciences), and CD34 APC (Clone 581, BD Biosciences). 7-amino actinomycin D (eBioscience) was added to cells as a cell viability marker. Cells were stained in primary antibodies for 45 minutes at 4^°^C. Antibodies were then washed from cells by centrifugation at 100 x g followed by resuspension in PBS with 0.1% BSA. MNC samples were then analyzed by flow cytometry using a BD FACSCalibur or Accuri C6 flow cytometer. Analysis of flow cytometry plots was performed using FCS Express (De Novo Software). Gating was performed to exclude 7-AAD+ cells in the FL-3 channel (R1), and low side scatter CD34+ cells (R2), and CD34+ Lineage- or CD45 dim+ (R3) (see Figure 1A).

### Cytokine and Chemokine Assays

Plasma levels G-CSF, GM-CSF, IL-17 and SDF-1 were using an ELISA kit (R&D Systems).

### Analysis of Cell Intrinsic Effects of LDL Cholesterol on isolated CD34+ HSPCs

CD34+ HSPCs were sorted from donor mononuclear cells by lineage depletion followed by CD34+ cell retention according to the manufacturer’s protocol (Miltenyi Biotec). Sorted CD34+ cells were then cultured under serum free conditions in StemSpan SFEM (Stem Cell Technologies) with recombinant IL-6, stem cell factor, thrombopoietin, and FLT3 ligand (100 ng/mL) each with StemRegenin 1 (1 μM, Cellagen Technologies) as described previously [14]. CD34+ HSPCs were expanded in vitro for 10 days. Cell quality was determined by multimarker flow cytometry analysis. Expanded CD34+ cells were treated with LDL cholesterol (100 ug/mL; Biomedical Technologies Inc.) for 48 hours and the number of CD34+ lineage- cells measured by flow cytometry. The number of CD34+ cells and mean fluorescence intensity of CXCR4 (Clone 12G5; eBioscience), GCSFR/CD114 (Clone LMM741; Miltenyi Biotec), VLA4/CD49d (Clone 9F10; eBioscience), CD47 (Clone 2D3; eBioscience) and CD34 was determined after exposure to LDL cholesterol as above.

### Statistical Analysis

Continuous variables are reported as mean ± standard deviation. Regression analyses were tested for significance using Pearson’s correlation. Comparisons between two treatment groups were analyzed using a t-test. Comparisons between statin therapies shown in Table 1 were tested using a two-way ANOVA with a Holm-Sidak post hoc test.

## Results

### Method of Quantification and Characterization of CD34+ Lineage- and CD34+ CD45dim Cells in Human Peripheral Blood Mononuclear Cells

Flow cytometry was used to quantify CD34 cells in adult human peripheral blood samples (Figure 1A). 7-AAD was used to exclude dead cells. We specifically selected low side scatter CD34+ cells that lacked lineage markers (CD3, 14, 16, 19, 20, and CD56 negative) and had dim CD45 expression as others have previously shown that this cell population contains hematopoietic progenitors [15]. Immunophenotyping of sorted CD34+ lineage- cells revealed the cells were negative for CD3, CD14, 16, 19, 33, CD45RA, and HLA-DR. Additionally, 16 ± 5.2% of the purified CD34+ lineage- cells were CD38-, 28 ± 5.2% were CD71+ and 0.87 ± 0.18% were CD90+. The findings support the CD34+ lineage- CD45 dim population of cells we aimed to quantify as having the immunophenotype of HPCs in human peripheral blood [15].

To verify that CD34+ lineage- cells were indeed a hematopoietic progenitor population, we performed hematopoietic colony formation assays in methylcellulose containing medium. Purified CD34+ Lineage- cells formed granulocyte, monocyte, and erythroid colonies, confirming their biological phenotype as precursors of the granulocyte, monocyte, and erythroid lineages (Figure 1B). Based on these findings we will refer to CD34+ cells as CD34+ hematopoietic stem/progenitors (HSPCs) through the remainder of the manuscript.

### Characteristics of Study Subjects

The aim of our study was to determine if serum cholesterol levels modulate the quantity of CD34+ HSPCs in the bloodstream of humans. We recruited subjects without known cardiovascular disease or diabetes mellitus (seven males, five females), average age 43.4 ± 12.5 years (Table 1). The subjects were healthy with an average body mass index of 24.9±7.2 kg/m^2^, and none smoked. The cohort also had a low cardiovascular disease risk factor profile with a mean systolic blood pressure of 119.5±35 mm Hg, total cholesterol 210.3±60.7 mg/dL, C-reactive protein levels 1.1 ± 1.3 mg/L and Framingham risk score 1.7±0.5 (Table 1). Baseline serum lipids revealed total cholesterol (average, 210.5 ± 27.6 mg/dL, range 168 to 282 mg/dL), LDL cholesterol (136.2 ± 22.9, range 114 to 205) levels spanned the normal and hypercholesterolemic range. Basal CD34+ Lineage- and CD34+ CD45dim cell numbers were measured as a percentage of total buffy coat mononuclear cells at 0.15 ± 0.1%.

### CD34+ HSPC Levels Vary with Serum Cholesterol Levels

Three different HMG-CoA reductase inhibitors or statins (atorvastatin 80 mg daily, pravastatin 80 mg daily or rosuvastatin 10 mg daily) of differing potency were given to study subjects to vary the total and LDL cholesterol levels and determine the relationship of these levels with CD34+ cell counts. All three statin therapies provided significant reduction in total and LDL cholesterol levels. As expected pravastatin was the least potent in reducing serum lipid levels, while atorvastatin most significantly reduced lipid levels (Table 1). We noted small but significant reductions in the levels of CD34+ Lineage- and CD34+ CD45dim (0.1 ± 0.06%) cell numbers in blood with statin therapy as indicated in Table 1. This finding suggested that serum lipid levels and the number of CD34+ HSPCs in the bloodstream might be linked.

**Figure 2:**
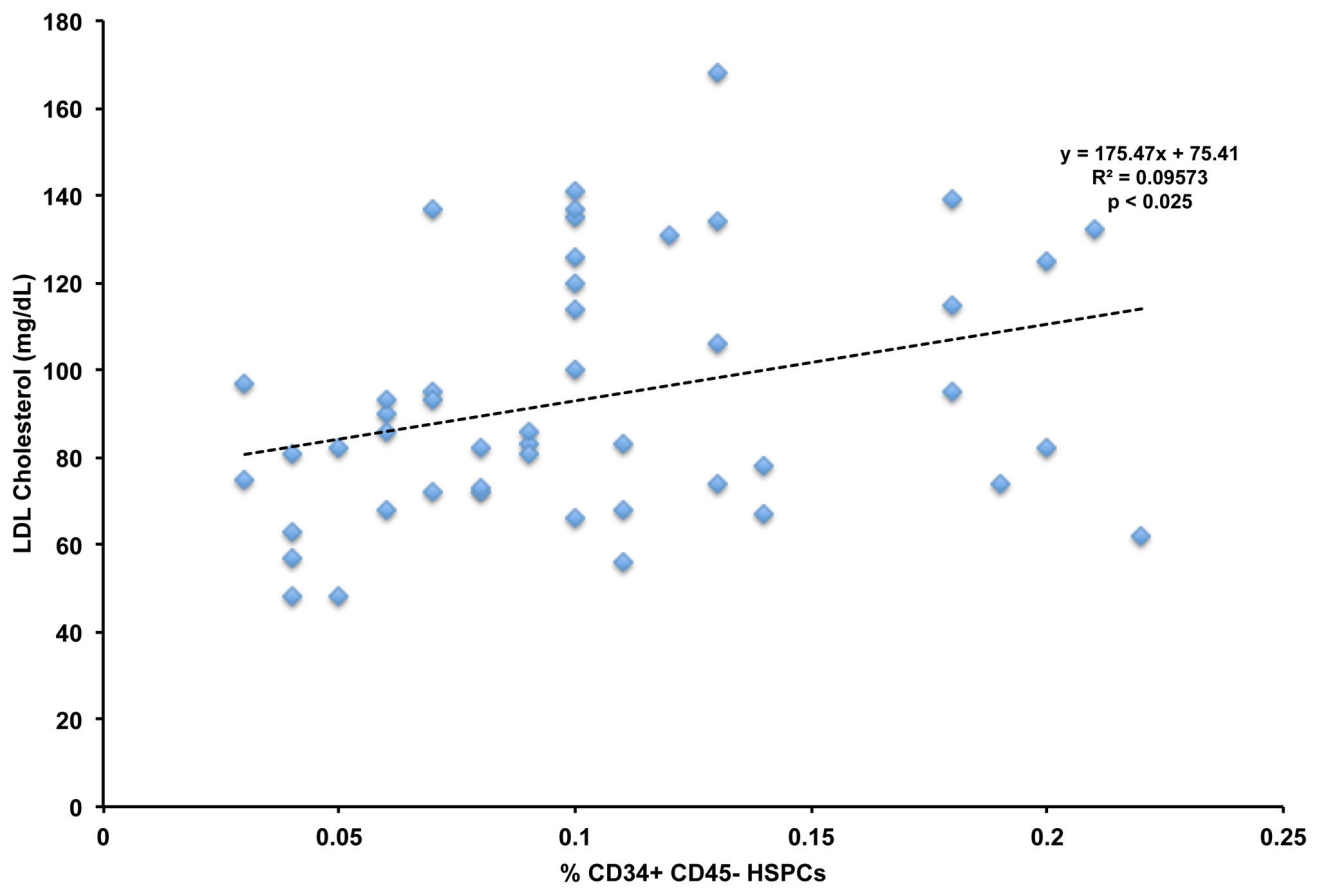
Correlation of LDL Cholesterol Levels and CD34+ HSPCs. LDL cholesterol and CD34+ HSPC numbers quantified across all subjects at baseline and after all statin treatments. p ≤ 0.025 determined by Pearson’s correlation.

To test the relationship between serum lipid levels and CD34+ HSPC numbers in blood, we performed regression analyses between CD34+ HSPCs and serum total, LDL, HDL cholesterol levels in all samples from twelve individuals at baseline and after three different statin treatments providing 48 data points. We found that CD34+ HSPCs and HDL cholesterol values did not correlate significantly with CD34+ HSPC numbers (r^2^=0.002). We did find a weak but significant positive correlation between total cholesterol and CD34+ HSPCs (r^2^=0.095, p < 0.025), and LDL cholesterol (r^2^=0.096, p < 0.025) shown in Figure 2. These findings suggest that LDL cholesterol modulates the quantity of CD34+ HSPCs in the bloodstream of humans.

### LDL Cholesterol Promotes Growth of Undifferentiated CD34+ HSPCs

**Figure 3:**
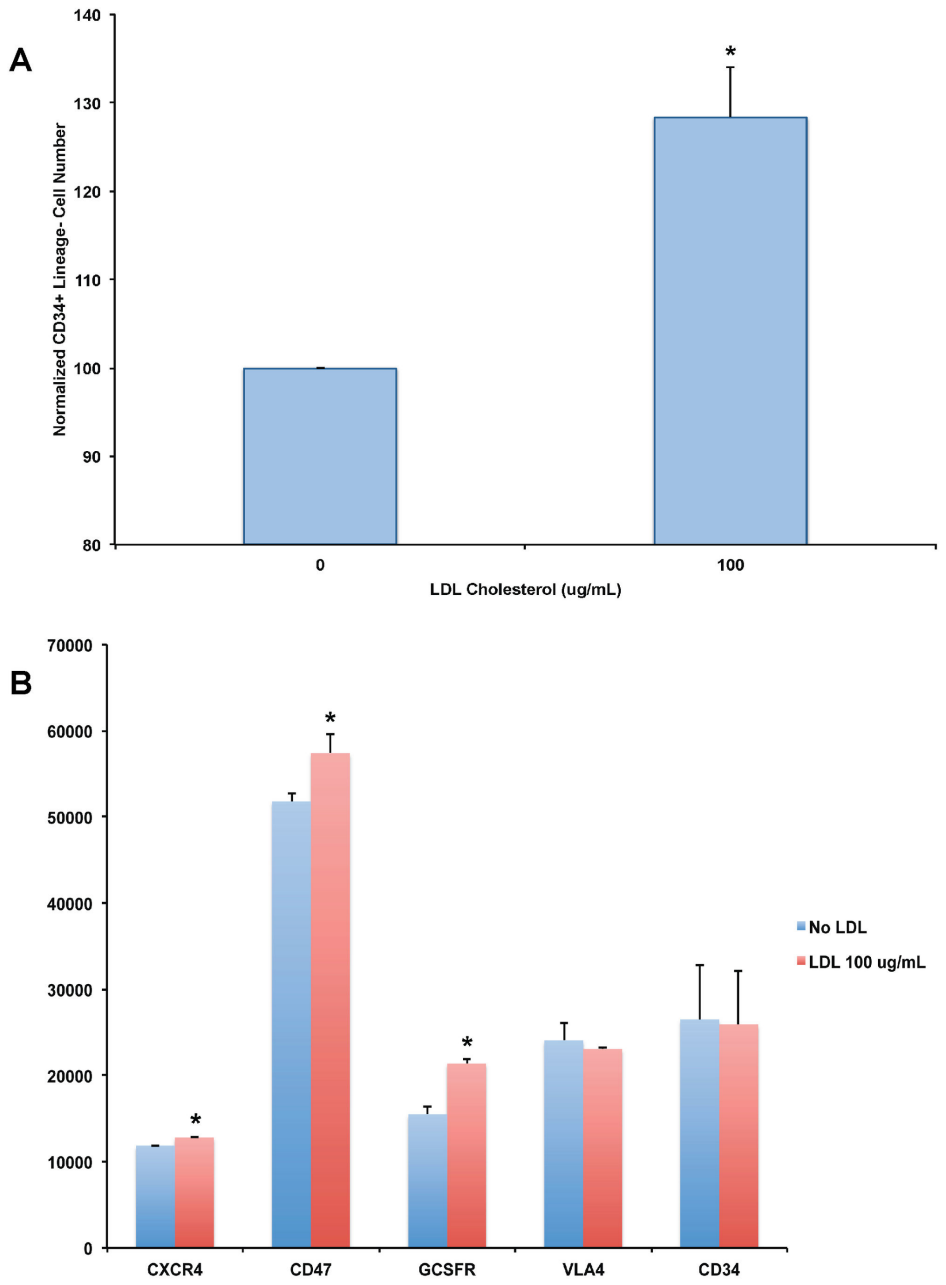
Effects of LDL Cholesterol of CD34+ HSPC proliferation and Surface Receptor Expression. Panel **A**: Sorted CD34+ HSPCs (n=6 individuals) were expanded in serum free medium with cytokines and Stemregenin 1, then exposed to no treatment or LDL cholesterol (100 ug/mL) for 48 hours and CD34+ Lin- low SSC cells were quantified by flow cytometry. * p < 0.03 vs. untreated cells. Panel **B**: Mean fluorescence intensity of CXCR4, CD47, GCSFR, VLA4 and CD34 determined from sorted CD34+ HSPCs after expansion either no treatment or LDL cholesterol (100 ug/mL); n=6 subjects, * p < 0.05.

Several potential mechanisms may be involved in regulation of CD34+ HSPC numbers in the bloodstream by LDL cholesterol. To begin to understand the possible interactions we first tested whether LDL cholesterol directly affects the growth of isolated CD34+ HSPCs. In this set of experiments we purified CD34+ lineage- cells from human peripheral blood. Then the cells were expanded in serum free medium with recombinant cytokines and a small molecule antagonist of the aryl hydrocarbon receptor StemRegenin 1 for 10 days [14] to study the growth of isolated CD34+ HSPCs in an undifferentiated state. Using this approach we obtained 85 ± 3.5% CD34+ CD38- cells. We then determined if LDL cholesterol affected the number of CD34+ cells. We found that LDL cholesterol (100 μg/mL) significantly increased the quantity of CD34+ lineage- cells 28 ± 5.7% (p < 0.03) after 48 hours of treatment compared with untreated cells (Figure 3A). The findings support an increase in CD34+ HSPC growth as one mechanism to explain the modulation of cell numbers in the bloodstream by cholesterol levels.

### Effects of LDL Cholesterol on CD34+ HSPC Cell Surface Molecules Involved in Mobilization and Protection from Phagocytosis

To further understand how LDL cholesterol levels modulate the quantity of CD34+ HSPCs in the bloodstream, we assessed how LDL cholesterol affects cell surface molecules involved in their migration and release from bone marrow (CXCR4 and VLA4), protection from phagocytosis (CD47) [16], and ability to respond to mobilization by G-CSF (GCSF receptor) as these to processes regulate the quantity of cells in the peripheral blood pool. Increased LDL cholesterol levels in vivo might act to reduce the expression of cell surface molecules that promote adherence of CD34+ HSPCs in the bone marrow niche, promoting their release into the circulation, increase expression of CD47 to protect them from phagocytosis, or increase GCSF receptor expression promoting their release from the niche into the peripheral blood pool. To test these concepts, purified CD34+ Lineage- HSPCs from adult human peripheral blood were expanded in vitro with StemRegenin 1 as described above. We then determined if the mean fluorescence intensity (MFI) of CXCR4, CD47, GCSFR, VLA4 and CD34 was affected by treatment of CD34+ HSPCs with LDL cholesterol (100 μg/mL) for 48 hours (Figure 3B). We found that exposure of CD34+ HSPCs to LDL cholesterol significantly increased the MFI of CXCR4 12800.3 ± 71.2 vs. 11847 ± 60.5 in untreated cells, p < 0.02, while VLA-4 MFI was not significantly affect by treatment with LDL cholesterol. CD47 expression significantly increased (57388 ± 2222 vs. 51796 ± 1019 untreated control, p < 0.05) after treatment with LDL cholesterol, indicating LDL cholesterol maintains CD47 expression in CD34+ HSPCs. G-CSF receptor expression also increased upon exposure to LDL cholesterol (21386 ± 561 vs. 15474 ± 871 in untreated control cells, p < 0.05), which potentially increases the sensitivity of CD34+ HSPCs to mobilization from the niche upon exposure to G-CSF. Finally, CD34 MFI was not significantly affected by LDL treatment. Overall, the findings support a model where LDL cholesterol increases the expression of CXCR4 a key receptor involved in cell migration, and the G-CSF receptor. Both cell surface molecules are likely involved to increase the peripheral blood CD34+ HSPC pool with increased serum lipid levels.

### LDL Cholesterol Increases Granulocyte and Monocyte Differentiation

**Figure 4:**
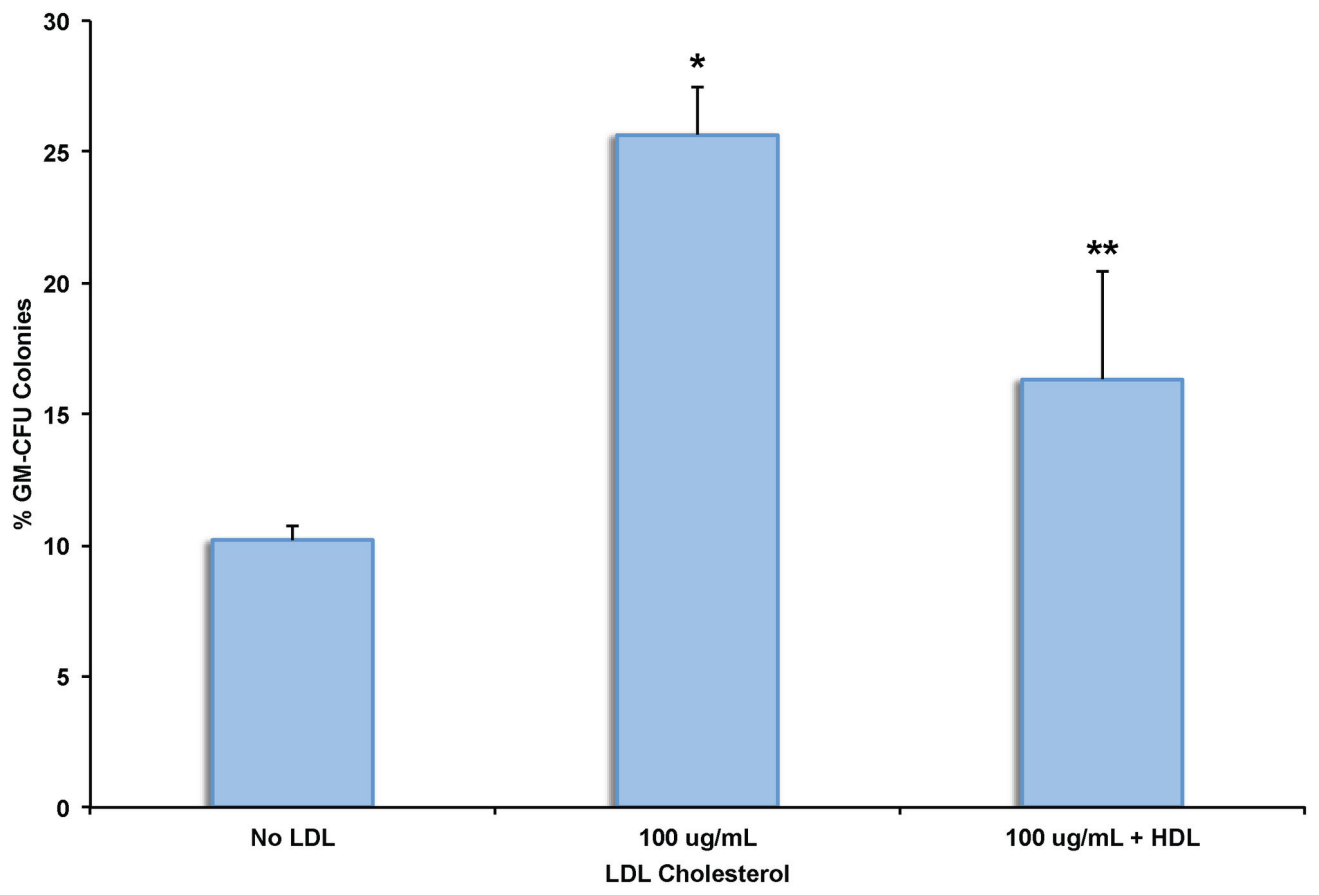
Effect of LDL cholesterol on Granulocyte and Monocyte Lineage Differentiation of CD34+ HSPCs. Sorted CD34+ HSPCs were plated in methylcellulose with cytokines with or without LDL cholesterol. Granulocyte Monocyte colonies were quantified by microscopy after 14 days in culture. n=6 subjects, * p < 0.05 vs. no LDL cholesterol, ** p < 0.05 vs. LDL cholesterol (100ng/mL).

Hypercholesterolemia in humans is associated with increased monocyte and neutrophil counts [3]. We next asked whether LDL cholesterol affects the differentiation of CD34+ HSPCs in methylcellulose-based assays. We found that LDL cholesterol significantly increased the number of granulocyte and monocyte colonies (25.7 ± 1.5 vs. 10.2 ± 0.6% for untreated CD34+ HSPCs, n= 6, p < 0.03), and was reversed by co-incubation with HDL cholesterol (Figure 4). The findings one mechanism to increase neutrophil and monocyte counts by LDL cholesterol as found in patients with hypercholesterolemia.

### Cholesterol Levels Positively Correlate with G-CSF and IL-17 Levels

**Figure 5:**
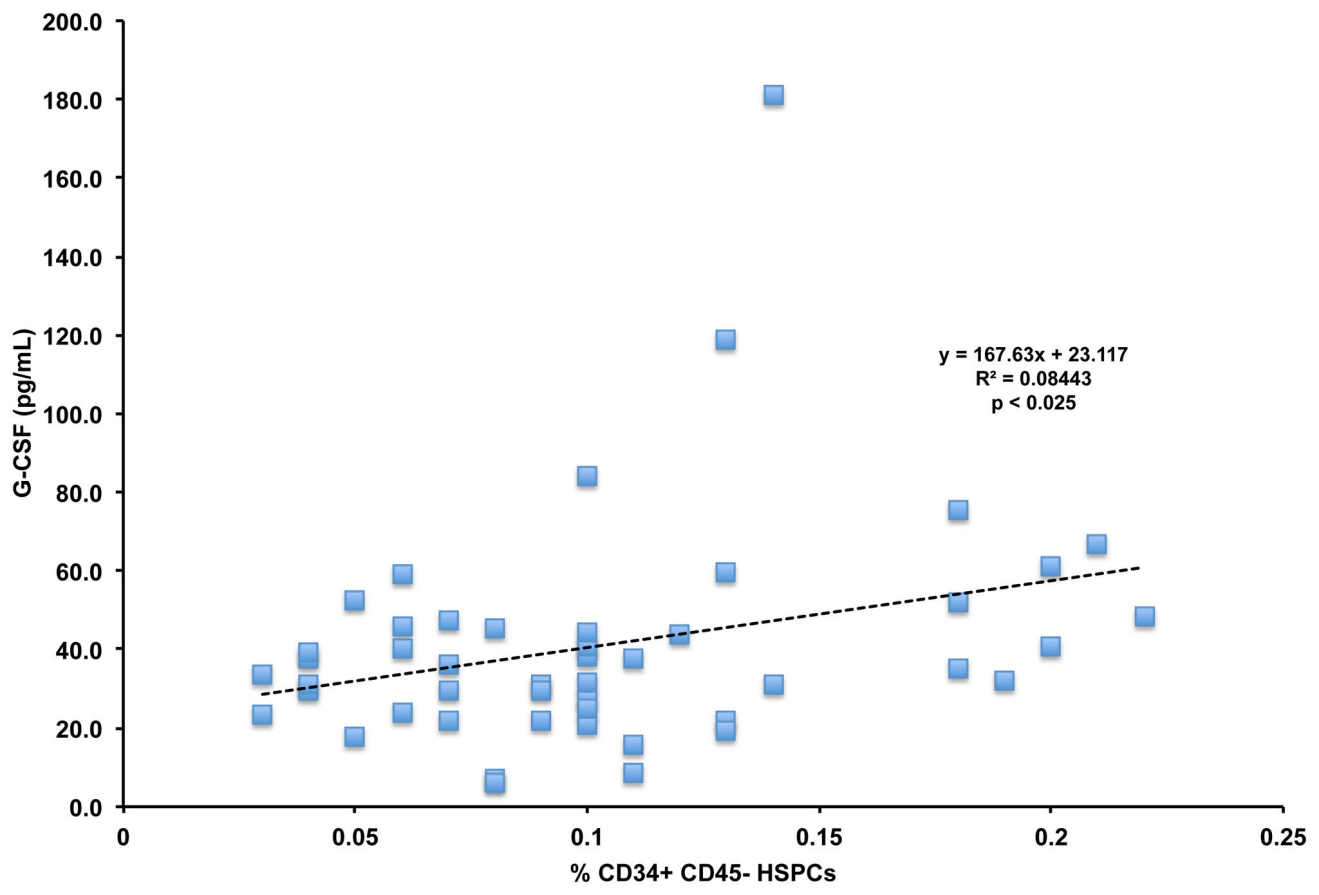
Correlation Between CD34+ HSPCs and G-CSF Levels. LDL cholesterol and G-CSF levels were determined across all subjects at baseline and after all statin treatments. p < 0.025 using Pearson’s correlation.

**Figure 6:**
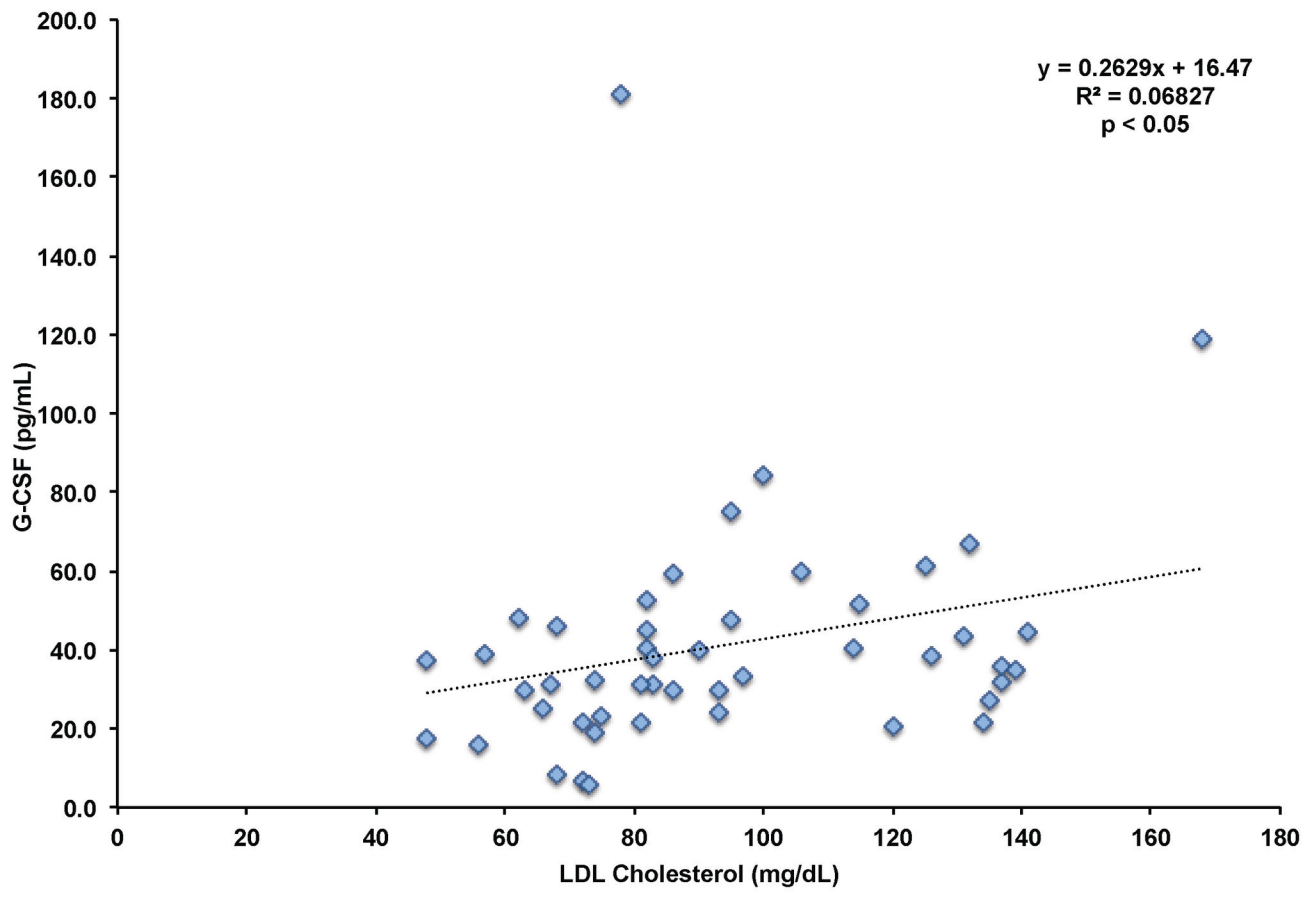
Correlation Between LDL Cholesterol and G-CSF Levels. LDL cholesterol and G-CSF levels were determined across all subjects at baseline and after all statin treatments. p < 0.05 using Pearson’s correlation.

To assess how cholesterol levels regulate the numbers of CD34+ HSPCs in the bloodstream, we measured the levels of the mobilizing cytokines G-CSF and SDF-1 in the plasma of our human subjects cohort. We found no significant correlation between SDF-1 levels and CD34+ HSPCs (r^2^=0.019). We did find a significant positive correlation between G-CSF levels and CD34+ HSPC numbers in the bloodstream (r^2^=0.084, p < 0.025) shown in Figure 5. We then determined if there was an interaction between cholesterol levels and G-CSF levels. We found significant positive correlations between G-CSF levels and total cholesterol (r^2^=0.05, p < 0.05), and LDL cholesterol (r^2^=0.07, p < 0.05), shown in Figure 6.

IL-17 plays a central role to facilitate production of G-CSF and regulate hematopoiesis [17]. A homeostatic feedback loop controlling granulopoiesis has been established known as the IL-23 IL-17 G-CSF axis [18]. When levels of neutrophils are low, macrophage and dendritic cells release IL-23, which acts on T cells to induce IL-17 production. Conversely, when neutrophil levels are replete within tissues, IL-23 secretion is suppressed, decreasing one stimulus for IL-17 production [18]. IL-17 acts on many cells of the body including bone marrow stromal [17], epithelial and endothelial cells, fibroblasts, and myeloid cells [19].

**Figure 7:**
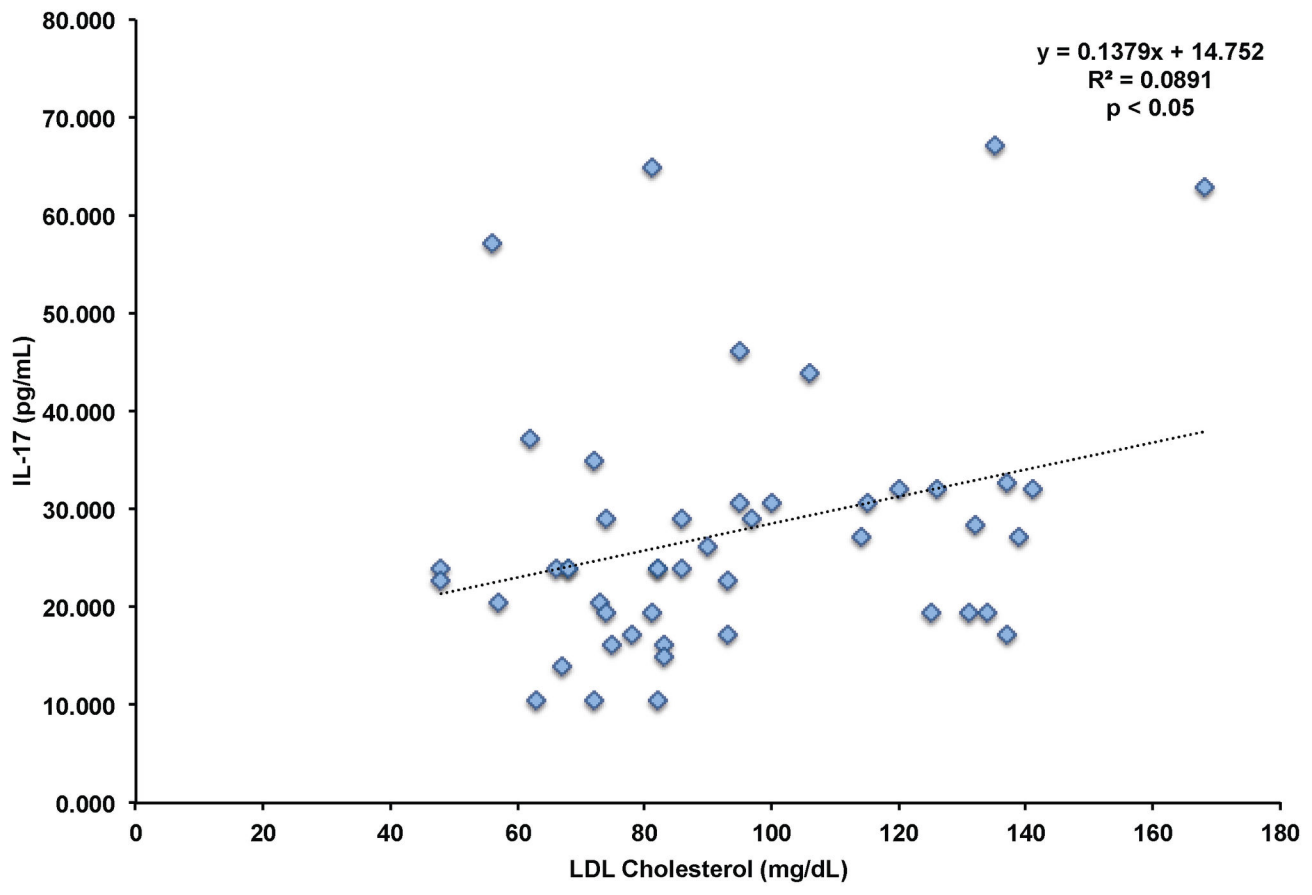
Correlation Between LDL Cholesterol and IL-17 Levels. LDL cholesterol and IL-17 levels were determined across all subjects at baseline and after all statin treatments. p < 0.05 using Pearson’s correlation.

In cholesterol efflux (ABCA1^-/-^ ABCG1^-/-^) knockout mice, the increased numbers of HSPCs in the bloodstream corresponded with increased IL-17 and G-CSF levels, and IL-17 and G-CSF neutralizing antibodies decreased HSPC levels in animals with disordered cholesterol homeostasis [11]. Given the role of IL-17 in regulation of G-CSF and hematopoiesis, and our finding that G-CSF correlated positively with CD34+ HSPC levels, we hypothesized that IL-17 may correlated with CD34+ HSPCs, total and LDL cholesterol levels. We found a weak positive correlation between CD34+ HSPC numbers and IL-17 levels that did not reach statistical significance (r^2^=0.046, p < 0.1). However, we did find that IL-17 levels positively correlated with both total cholesterol (r^2^=0.082, p < 0.05) and LDL cholesterol levels (r^2^=0.089, p < 0.05) shown in Figure 7. The findings support a model where LDL cholesterol regulates IL-17 levels, controlling G-CSF levels, which modulate the mobilization of CD34+ HSPCs in the bloodstream. The effect of altered cholesterol metabolism on the IL-17/G-CSF axis, and HSPC mobilization are supported by similar findings in ABCA1^-/-^ ABCG1^-/-^ in vivo [11].

## Discussion

The findings of this hypothesis generating study support the following conclusions: 1) The quantity of circulating CD34+ CD45dim Lineage- HPCs are decreased by cholesterol reduction with statins, 2) The quantities of circulating HPCs in humans positively correlate with total and LDL cholesterol levels, 3) LDL cholesterol increases the proliferation of CD34+ HSPCs, and augments the expression of cell surface molecules involved in adherence and migration, and protection from phagocytosis 4) LDL cholesterol levels positively correlate with the levels of IL-17, and the CD34+ HSPC mobilizing cytokine G-CSF. While our study cohort was small and did not include subjects with known coronary artery disease but remarkably do correspond with similar studies in animal models of hypercholesterolemia [8,9] or disordered cholesterol metabolism [10]. Given the prior findings in mice and in humans [8] that elevated cholesterol levels increase the quantity of neutrophils and platelets in blood, the finding that HPC levels fall with reduction in cholesterol levels is a conceptually related but important finding because HPCs generate neutrophils, monocytes and macrophages, the primary cellular mediators of atherosclerosis. HSPC mobilization also plays an important role in contributing to increased splenic monocyte differentiation and acceleration of atherosclerosis in response to acute myocardial infarction [6], reinforcing the role of circulating HSPCs in atherosclerosis and the importance of understanding how modifiable atherosclerosis risk factors such as cholesterol levels modulate their numbers and activity.

We determined that in human subjects without known cardiovascular disease that the quantity of CD34+ HSPCs in the bloodstream positively corresponds with plasma LDL concentration. We determined that LDL cholesterol increases the expression of CD47, a cell surface molecule that protects HSPCs from phagocytosis, or expression of cell adhesion molecules CXCR4 or VLA-4, both required for maintenance of adhesion in the bone marrow niche. Additionally, we found no effect of varying LDL cholesterol levels on the quantity of SDF-1 in the bloodstream, indicating that the measurable plasma concentration of SDF-1 and the HSPC derived adhesion factors are not decreased by cholesterol concentration to explain the modulation of HSPC levels in blood by cholesterol levels. Studies in the ABCA1/ABCG1^-/-^ knockout mouse indicated knockout of cholesterol efflux transporters led to a reduction in osteoblast SDF-1 levels [10]. Effects of cholesterol levels on expression of SDF-1 in the bone marrow niche, or on osteoblast quantity are possible additional mechanisms to explain our observations on HSPC numbers in the bloodstream, but cannot be assessed in human subjects.

We did find that the hematopoietic cytokine G-CSF, but not GM-CSF, corresponded to the quantity of HSPCs in the bloodstream. Overall the finding that LDL cholesterol levels modulate the quantity of HSPCs in the bloodstream in humans is a novel finding, and is well supported by recent publications in the field in mouse models. A growing number of manuscripts in murine models of hypercholesterolemia show that increasing serum cholesterol levels either through dietary fat intake or in combination with LDLR^-/-^ increases the quantity of HSPCs in the bloodstream, in line with our findings. Our study found less substantial variation in CD34+ HSPC levels over a clinically relevant range of cholesterol values. This is most likely due to significantly higher cholesterol values obtained in LDLR^-/-^ mice fed a high fat diet. Importantly, the total and LDL cholesterol values in our human cohort well reflects the cholesterol values found in studies of human subjects with acute coronary syndromes [12].

The effects of elevated cholesterol on CD34+ HSPC mobilization are potentially mediated by a combination of effects on HSPC proliferation, and on on IL-17 and G-CSF levels. In the ABCA1/ABCG1^-/-^ cholesterol efflux mutant mouse model, elevated numbers of HSPCs were found in the blood through a complex mechanism that in part involved increased levels of IL-17 which in turn regulated increased G-CSF levels to mobilize HSPCs. We found positive correlations between CD34+ HSPC numbers and G-CSF levels, and between LDL cholesterol levels and IL-17 levels. The molecular mechanism involved in regulation of IL-17 levels by cholesterol levels remains to be determined, but may involve either a secondary effect of cholesterol to modulate the cytokines required to induce the T_H_17 T cell fate (IL-6, IL-21, and TGFβ) [20], or a direct effect of cholesterol as a ligand for the required IL-17 inducing transcription factor RORγt in the production of IL-17 [21,22]. Future studies may explore the interaction of IL-17 and cholesterol, and determine if therapies to modulate either the differentiation of T_H_17 cell, IL-17 production or activity has any impact on cardiovascular disease events. Modulation of IL-17 levels and HSPC levels in patients with heart disease may prove useful as an adjunct to standard preventative therapies including statins, or as an alternative to statins in patients who are intolerant to these medications. Our finding that circulating HSPC levels increase with total and LDL cholesterol levels also call for a hypothesis testing study to determine if HSPCs, like cholesterol levels, are linked to CVD events.
